# Opportunities, challenges, and future directions of large language models, including ChatGPT in medical education: a systematic scoping review

**DOI:** 10.3352/jeehp.2024.21.6

**Published:** 2024-03-15

**Authors:** Xiaojun Xu, Yixiao Chen, Jing Miao

**Affiliations:** Division of Hematology/Oncology, Children’s Hospital, Zhejiang University School of Medicine, National Clinical Research Centre for Child Health, Zhejiang, China; Hallym University, Korea

**Keywords:** Artificial intelligence, Data accuracy, Medical students, Medical education, Attention

## Abstract

**Background:**

ChatGPT is a large language model (LLM) based on artificial intelligence (AI) capable of responding in multiple languages and generating nuanced and highly complex responses. While ChatGPT holds promising applications in medical education, its limitations and potential risks cannot be ignored.

**Methods:**

A scoping review was conducted for English articles discussing ChatGPT in the context of medical education published after 2022. A literature search was performed using PubMed/MEDLINE, Embase, and Web of Science databases, and information was extracted from the relevant studies that were ultimately included.

**Results:**

ChatGPT exhibits various potential applications in medical education, such as providing personalized learning plans and materials, creating clinical practice simulation scenarios, and assisting in writing articles. However, challenges associated with academic integrity, data accuracy, and potential harm to learning were also highlighted in the literature. The paper emphasizes certain recommendations for using ChatGPT, including the establishment of guidelines. Based on the review, 3 key research areas were proposed: cultivating the ability of medical students to use ChatGPT correctly, integrating ChatGPT into teaching activities and processes, and proposing standards for the use of AI by medical students.

**Conclusion:**

ChatGPT has the potential to transform medical education, but careful consideration is required for its full integration. To harness the full potential of ChatGPT in medical education, attention should not only be given to the capabilities of AI but also to its impact on students and teachers.

## Graphical abstract

[Fig f5-jeehp-21-06]

## Introduction

### Rationale

The ChatGPT, launched in November 2022, is a large language model (LLM) based on artificial intelligence (AI). Trained on extensive text datasets in multiple languages, it possesses the capability to generate human-like responses [1]. Since ChatGPT came out, the scientific community’s opinions have been mixed. On the one hand, ChatGPT helps to improve efficiency in academic writing [2-4]. On the other hand, it is limited by its training datasets, leading to seemingly reasonable yet erroneous outputs [5,6]. Other potential concerns include privacy breaches and the dissemination of misinformation [5,7,8]. In the healthcare domain, ChatGPT has demonstrated significant value, aiding in clinical diagnosis and decision-making, the provision of personalized healthcare, drug development, and the analysis of large clinical datasets [9,10]. However, its applications in medical education have received limited exploration despite its vast potential. Given the substantial amount of information and concepts that medical students need to grasp, this area is interesting and worthy of exploration.

### Objectives

This paper conducted a scoping review of existing literature discussing ChatGPT in the context of medical education, extracts key points regarding the advantages and disadvantages of ChatGPT in medical education. We also aim to provide a foundation for future research and offer feasible insights and evidence for further exploration in this domain.

## Methods

### Ethics statement

This was a literature-based study; therefore, neither approval from the institutional review board nor informed consent was required.

### Study design

This study conducted a scoping review, described in accordance with the Preferred Reporting Items for Systematic Reviews and Meta-Analyses Extension for Scoping Reviews (PRISMA-ScR) guidelines [11].

### Protocol and registration

An internal review protocol was developed, but was neither registered nor published (Supplement 1).

### Eligibility criteria

Our primary research questions were: what are the potential benefits and limitations of ChatGPT in medical education, and what are the future directions? We aimed to guide future research by searching the literature on the application of ChatGPT in medical education, delineating its potential application value, and assessing challenges and limitations.

Inclusion criteria: articles or preprints discussing ChatGPT in the context of medical education; written in English; and, published between January 1, 2022 and November 30, 2023. Exclusion criteria: non-English writing; articles focusing solely on non-clinical medical education (e.g., nursing, pharmacy, and dentistry); and articles unrelated to medical education.

### Information sources and search

The databases included PubMed/MEDLINE, Embase, and Web of Science. As ChatGPT gained widespread acceptance and application after 2022, the search timeframe was limited from January 1, 2022, to November 30, 2023. The search statement can be found in Supplement 2. Two reviewers independently conducted a systematic search.

### Selection of sources of evidence

Article selection was independently conducted by 2 authors, and discrepancies were resolved through independent review by a third author (J.M.). A final consensus was reached through author meetings.

**Fig. 1.**
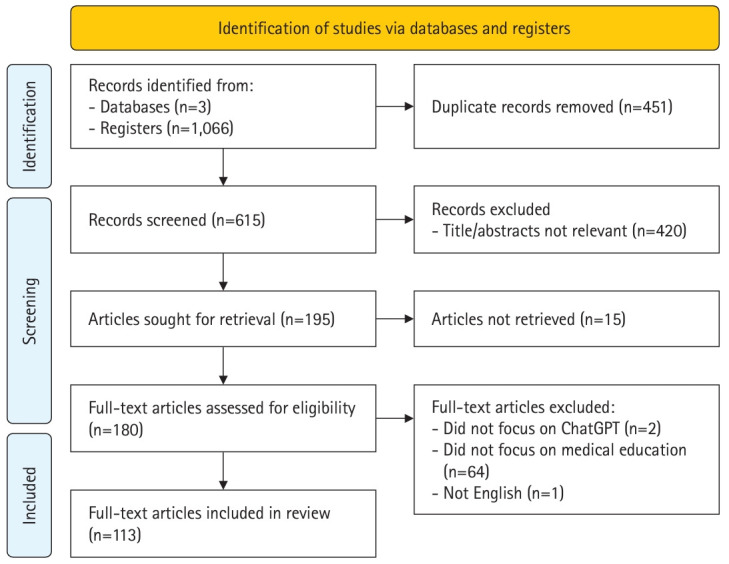
The flow diagram of searching and screening for articles on ChatGPT in medical education.

The search results from PubMed/MEDLINE, Embase, and Web of Science were imported into EndNote X9 (Clarivate), generating a total of 1,066 records. Initially, 451 duplicate records were excluded, followed by title and abstract screening, resulting in the exclusion of 420 irrelevant articles. Subsequently, full-text screening was performed on the remaining 195 articles, with 15 articles excluded due to unavailability of full texts. Additionally, 2 articles did not focus on ChatGPT, 64 articles solely addressed non-physician education, and one article was not in English, resulting in the inclusion of 113 articles (Fig. 1).

### Data charting process and data items

A specialized search was conducted for each included article, extracting the following information: article type (preprint, research article, review, commentary, etc.); potential applications and benefits of ChatGPT in medical education; potential risks and limitations of ChatGPT in medical education; and suggestions on the application of ChatGPT in medical education.

### Critical appraisal of individual sources of evidence

The primary emphasis of the research is on a comprehensive scoping review rather than an in-depth analysis of individual sources of evidence. In order to maintain overall coherence and thematic consistency in the study, the decision was made to forego a detailed evaluation of individual sources of evidence.

### Synthesis of results

Thematic analysis was conducted of the extracted data. Initially, open coding was performed on the content in the extraction table, followed by the creation of axial codes to categorize existing codes. The data were then recoded into primary and secondary themes decided through discussion. We focused on the potential applications and limitations of ChatGPT in medical education and related suggestions (Supplement 3).

## Results

### Selection of sources of evidence

As shown in Fig. 1, we initially identified 1,066 records through database searches, and after comprehensive screening, a total of 113 articles were included.

### Characteristics of the sources of evidence

The majority of articles (101/113, 89.4%) mentioned the potential applications or benefits of ChatGPT in medical education. Furthermore, 61.9% of the articles (70/113) mentioned the potential risks and limitations of ChatGPT in medical education. Regarding the types of articles, 37.2% (42/113) of records were original research articles.

### Critical appraisal within sources of evidence

The primary focus of this review was to provide a comprehensive overview of existing literature and to synthesize information and present a broader understanding of the topic, rather than conducting an in-depth critical appraisal of individual sources. Therefore, a critical appraisal of sources of evidence was not done.

### Results of individual sources of evidence

The relevant data from the included studies are summarized in Supplement 4.

### Synthesis of results

#### Potential applications and benefits of ChatGPT in medical education

##### Enabling novel learning approaches through ChatGPT

A substantial amount of literature emphasized the enormous potential of ChatGPT in assisting students in acquiring medical knowledge and problem-solving. Students can ask ChatGPT specific medical questions and swiftly obtain accurate and personalized answers to help them build their knowledge base [12]. ChatGPT’s powerful capabilities of information collection and summarization can improve the efficiency of students’ knowledge retrieval, simplify the learning process, save time, and allow better focus on learning [13-15]. Additionally, ChatGPT is convenient to use and instant to access. It can support medical students’ learning through mobile applications [16].

**Fig. 2.**
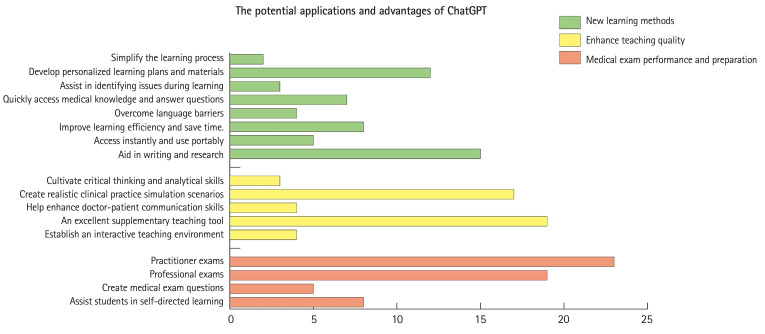
Summary of potential applications and advantages of ChatGPT based on the included records.

Many articles also highlighted the significant potential of ChatGPT in meeting the personalized needs of learners, providing a personalized learning experience [17]. Developing personalized learning plans and learning materials, as well as providing tailored feedback to learners, are potential application avenues to explore [18]. Moreover, several articles discussed the use of ChatGPT as a potential writing or research assistant [19]. ChatGPT not only holds great potential in assisting with literature reviews and summaries [20], but it can also help non-native English speakers improve their writing skills and provide comprehensive translations of foreign-language content [21] (Fig. 2, Supplement 3).

##### Improving teaching quality through ChatGPT

The potential application of ChatGPT for improving teaching quality has been most frequently mentioned is creating realistic clinical simulation scenarios for medical students [22,23]. It not only aids medical students in transitioning quickly from pre-clinical to clinical states [24], but also provides a safe and controlled environment for practicing clinical skills [17,22]. Simulated scenarios can be used as in-class tests as a time-efficient way of evaluating students’ abilities [17,19] and addressing the shortage of standardized patients [25]. Given ChatGPT’s interactive capabilities, its enormous potential is foreseeable in assisting medical students in improving doctor-patient communication skills, helping to improve communication skills [26].

A significant number of articles emphasized the substantial value of ChatGPT for application as an auxiliary teaching tool [17,22,23,27]. ChatGPT can be used for innovating teaching methods, such as flipped classrooms and problem-based learning [28], aiding in the development of curricula and teaching plans [23], establishing interactive teaching environments [27], and even serving as a virtual assistant to reduce teachers’ workload [29,30] (Fig. 2, Supplement 3).

##### Medical exam performance and exam preparation with ChatGPT

Several studies focused on ChatGPT’s performance in medical knowledge tests, including licensing examinations for physicians, anesthesia, ophthalmology, neurology, and other specialty examinations [31-34]. Overall, ChatGPT demonstrated passing scores in most countries’ licensing and specialty exams, but generally scored only slightly above the passing line, and did not achieve accuracy rates above 95% in any licensing exam. Some studies investigated ChatGPT’s performance on different types of questions, revealing poorer performance in advanced judgment and multiple logical inference questions [35].

Some scholars believe that ChatGPT can be applied to self-directed learning and exam preparation, such as helping students review, facilitating group learning, and creating exam simulation questions [31,32,36,37] (Fig. 2, Supplement 3).

#### Potential risks and limitations of ChatGPT in medical education

##### Academic integrity and ethical issues

**Fig. 3.**
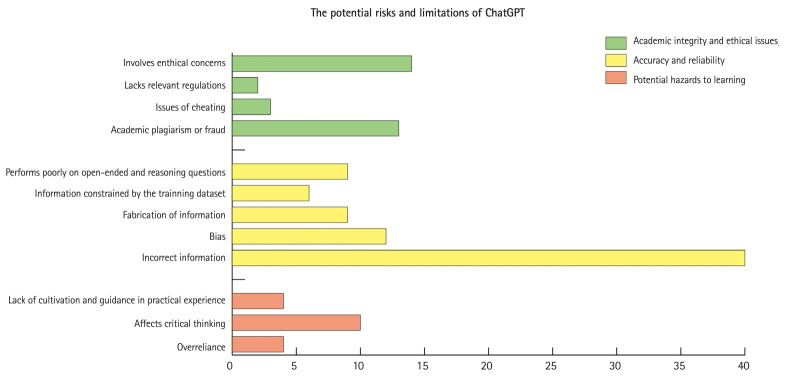
Summary of the potential risks and limitations of ChatGPT based on the included records.

Numerous scholars expressed concerns about potential threats to academic integrity posed by ChatGPT and its potential misuse [22,28,38]. Many potential advantages of ChatGPT can also be potential avenues for unethical behavior. For example, ChatGPT may be used for cheating in exams to get higher scores [16]. Students might plagiarize content generated by ChatGPT in their papers, affecting their critical thinking abilities and academic integrity [5]. Additionally, ChatGPT may pose potential threats to ethical issues [22,39]. ChatGPT may trigger issues related to data privacy, patient privacy, student and teacher privacy, intellectual property, and so forth [13,22,39], and some scholars even proposed the possibility of bioweapon creation and reinforcement of authoritarian regimes [40]. Currently, there is a lack of specific regulations or guidelines to guide the use of ChatGPT [13] (Fig. 3, Supplement 3).

##### Issues of accuracy and reliability

Issues related to ChatGPT’s accuracy and reliability were detailed in many articles, with 48 articles (42.5%) stating that ChatGPT may generate incorrect information and facilitate the spread of misinformation, including but not limited to providing incorrect or controversial medical advice, inaccurately explaining medical concepts, low accuracy rates, unspecified citations, lack of consistency, and generating seemingly reasonable but incorrect answers [5,28,39]. Several authors emphasized that ChatGPT’s knowledge base is limited by its training data and cannot provide the latest information [28,41]. Furthermore, ChatGPT performs poorly on open-ended and multiple logical inference questions [42].

Additionally, ChatGPT may fabricate information, and it is challenging to identify when it generates fabricated information [43]. Moreover, ChatGPT may have potential algorithmic biases, leading to discriminatory behavior and stereotypes, potentially resulting in unfair treatment of certain groups and perpetuating existing inequalities in the healthcare system [28,39] (Fig. 3, Supplement 3).

##### Potential harms to learning

Some literature pointed out the adverse effects on the learning process due to ChatGPT. Over-reliance on ChatGPT may hinder the cultivation of critical thinking and clinical reasoning abilities in medical students [44,45]. Moreover, an excessive emphasis on AI-based learning opportunities may reduce interpersonal interaction and engagement, which are foundational for learning and honing practical skills [46]. In addition, ChatGPT exhibits varying degrees of proficiency in different language environments, with its best performance in handling English texts but still facing challenges when dealing with non-English questions [41] (Fig. 3, Supplement 3).

#### Recommendations for medical students and teachers

##### Recommendations for medical students

**Fig. 4.**
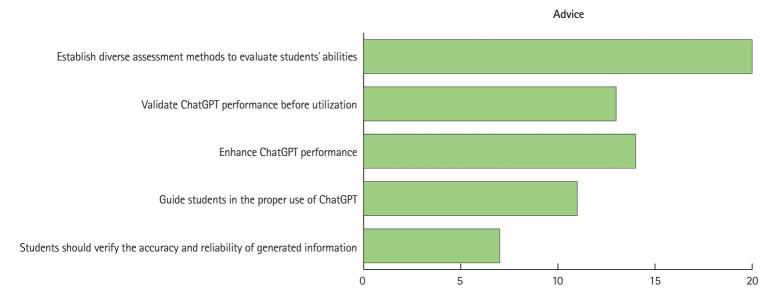
Summary of advice for medical students and teachers based on the included records.

Due to the potential risks and limitations of ChatGPT, many scholars advise medical students to use ChatGPT cautiously and verify the accuracy and reliability of generated information, such as cross-referencing with textbooks [37]. Students should use ChatGPT in an ethical and secure manner and disclose the use of AI-generated content in academic work (Fig. 4, Supplement 3).

##### Recommendations for teachers

Many articles emphasized that teachers should instruct students on how to use ChatGPT, including informing them of the limitations and advantages of AI, guiding them on how to discern the feasibility, authenticity, and accuracy of information provided by AI, and adhering to ethical and moral standards [47,48]. Before using ChatGPT for teaching assistance or applications, teachers must verify its safety, reliability, and repeatability and assess its impact on the content and quality of teaching to prevent adverse effects on the teaching process [39,48]. Moreover, considering the impact of ChatGPT on traditional assignments and assessments, it is recommended that teachers establish diverse assessment methods to evaluate students’ abilities, such as using presentations, practical assessments, and face-to-face exams [39,48].

Currently, the use of ChatGPT is mainly constrained by its accuracy and reliability issues. Some scholars suggest augmenting ChatGPT’s capabilities, such as addressing algorithmic biases, expanding the training dataset, improving its proficiency in different language environments, and increasing the consistency of responses [41,49] (Fig. 4, Supplement 3).

## Discussion

### Summary of evidence

ChatGPT, as a novel AI technology, is in a prevailing trend of popularization and applications in medical education. However, this trend has also brought numerous challenges. Understanding how ChatGPT may contribute to medical education is crucial for conducting in-depth research and optimizing its role in this context.

In this review of the latest research on ChatGPT in medical education, we have outlined its advantages and limitations. However, these factors are not independent but interact with each other, potentially amplifying or diminishing their impacts. For instance, ChatGPT can assist in constructing realistic clinical simulation scenarios, enhancing teaching quality, and improving students’ practical skills. Nonetheless, if errors from ChatGPT are introduced during this process, it may lead to the failure of teaching activities and even jeopardize patients’ safety. Moreover, synergies exist among ChatGPT's advantages. For example, medical textbooks, considered the gold standard for medical knowledge, have limitations such as being outdated and potentially containing inaccuracies [50]. Leveraging ChatGPT’s writing capabilities to synthesize the latest medical research into timely educational content can help students stay up-to-date with the latest developments.

### Limitations

This article has certain limitations that should be considered when interpreting the current review results. Firstly, the literature search was restricted to articles published in English, potentially excluding some relevant non-English literature, leading to selection bias. Secondly, documents that were inaccessible were excluded, which, although in small numbers, could result in missing relevant data. Given that the search for this review concluded on November 30, 2023, and literature on the application of ChatGPT in medical education is rapidly growing, further research and reviews are necessary.

### Suggestion

Future research should delve into the complex dynamic relationships between the advantages and limitations of ChatGPT in medical education. A more detailed examination of the interplay between these aspects will contribute to realizing the potential of ChatGPT in medical education and proactively addressing associated risks. Based on this, we propose 3 future research directions: first, cultivating the ability of medical students to use ChatGPT correctly; second, integrating ChatGPT into teaching activities and processes; and third, proposing standards for the use of AI by medical students.

### Cultivating the ability of medical students to use ChatGPT appropriately

As the use of ChatGPT continues to become more widespread, the most relevant challenge for medical students is the ability to use AI, which involves understanding the strengths and limitations of AI, critically evaluating generated information, and using AI responsibly [5,19,22,48]. While many articles emphasize the importance of guiding medical students in developing these skills, there is currently a lack of dedicated courses specifically tailored to ChatGPT.

Developing courses related to the use of ChatGPT for medical students is crucial. An essential aspect of these courses should be assisting medical students in dealing with potential inaccuracies and unreliability in ChatGPT-generated content. ChatGPT may generate erroneous and fabricated information, and its knowledge is limited to the training dataset [5,48,49]. Furthermore, the inaccuracy of AI can be improved, but not completely eliminated. As inaccuracies are still present in medical textbooks, the gold standard of medical knowledge [50], information generated by ChatGPT based on existing knowledge cannot completely eliminate those errors [51]. Therefore, helping medical students cope with potential inaccuracies and unreliability in ChatGPT-generated content should involve at least 2 aspects. Firstly, students should be helped to develop the ability to assess the accuracy and quality of information from any source. Evaluating the accuracy and quality of information may be a new challenge, but fundamentally, it should be similar to the previous assessment of the quality of medical literature, involving assessments of author credibility, source evaluation, and external reviews. However, ChatGPT does not provide citation sources, leading to a new challenge. Secondly, medical students should be instructed on how to draw correct conclusions in situations of data misinformation, absence, or inaccuracy.

### Integrating ChatGPT into teaching activities and processes

ChatGPT has the potential to create realistic clinical simulation scenarios and build interactive teaching environments; therefore, it can be applied in various innovative teaching methods [22,39,52]. While this could revolutionize medical education, careful consideration is necessary to determine whether these changes are beneficial for clinical teaching rather than solely focusing on efficiency or economic benefits. For example, using ChatGPT in clinical simulation scenarios can help medical students transition rapidly from pre-clinical to clinical states, alleviating shortages of standardized patients. However, it must be acknowledged that the excessive use of ChatGPT in medical education may hinder the development of medical students’ critical thinking and clinical reasoning skills [17,28,38], potentially impairing their practical abilities [38], which could pose a threat to patient safety. Therefore, any AI medical teaching program should undergo rigorous validation and assessment before widespread implementation, with research conducted in controlled and real-world learning scenarios [31].

### Establishing guidelines for the use of AI

Numerous articles express concerns about the potential risks of ChatGPT regarding academic integrity and ethical issues, including plagiarism, cheating on exams, privacy breaches, and damage to intellectual property [28,39,48]. Instances already exist where AI has been used to generate summaries and academic papers [53,54]. Therefore, there is an urgent need to establish guidelines for the use of ChatGPT in medical education. These guidelines should encompass accountability systems, ethical considerations, privacy, and moral and integrity issues [55]. Scholars have proposed the incorporation of 4 major ethical principles into the integration of AI into medical education: autonomy, fairness, non-malfeasance, and beneficence. However, specific guidelines for the use of AI still require further research.

## Conclusion

The transformative potential that ChatGPT brings to medical education is undeniable, yet its complete integration into medical education requires further exploration and in-depth consideration. While existing literature theoretically speculates on the prospects of ChatGPT in medical education, there is still a lack of sufficient empirical research to guarantee its effectiveness and rationality in medical education. Therefore, further research needs to be conducted on ways of cultivating medical students’ ability to use ChatGPT correctly, integrating ChatGPT into teaching activities and processes, and establishing guidelines for the use of AI. To unleash the maximum potential of ChatGPT in medical education, attention needs to be directed not only toward the capabilities of AI but also toward its impact on students and educators themselves.

## Figures and Tables

**Figure f5-jeehp-21-06:**
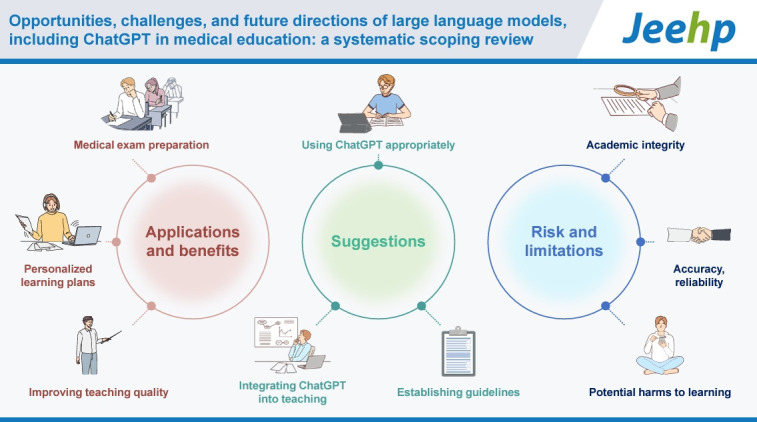


## Supplementary materials

Supplementary files are available from Harvard Dataverse: https://doi.org/10.7910/DVN/OXK5VE

Supplement 1. The internal review protocol.

Supplement. 2. Search queries terms in PubMed, Web of Science, and Embase for articles or preprints discussing on ChatGPT in the context of medical education, written in English, and published between January 1, 2022 and November 30, 2023.

Supplement 3. Primary theme, sub-themes, representative quotations, and relevant papers from the 113 included articles.

Supplement 4. The list of 113 included papers.

Supplement 5. Audio recording of the abstract.
