# Supplementary material for: Opportunities, challenges, and future directions of large language models, including ChatGPT in medical education: a systematic scoping review
Source: J Educ Eval Health Prof. 2024 Mar 15;21:6. doi: 10.3352/jeehp.2024.21.6 (PMC11035906; doi:10.3352/jeehp.2024.21.6)
Supplement: Supplementary file 1 — Supplement 1. The internal review protocol. [file jeehp-21-06-suppl1.docx]

**Supplement 1.** An internal review protocol

**Background**

The ChatGPT, launched in November 2022, is a large language model based on artificial intelligence (AI). Trained on extensive text datasets in multiple languages, it possesses the capability to generate human-like responses [1]. Since ChatGPT came out, the scientific community’s opinions have been mixed. On the one hand, ChatGPT helps to improve efficiency in academic writing [2]. On the other hand, it is limited by the training datasets, leading to seemingly reasonable yet erroneous outputs [3]. Additionally, potential concerns include privacy breaches and the dissemination of misinformation [3]. In the healthcare domain, ChatGPT has demonstrated significant value, aiding in clinical diagnosis and decision-making, providing personalized healthcare, assisting in drug development, and analyzing large clinical datasets [4]. However, its applications in medical education have received limited exploration despite the vast potential. Given the substantial amount of information and concepts that medical students need to grasp, this area proves to be interesting and worthy of exploration.

So, we conduct a scope review of existing literature discussing ChatGPT in the context of medical education, extracts key points regarding the advantages and disadvantages of ChatGPT in medical education. And we aim to provide a foundation for future research and offer feasible insights and evidence for further exploration in this domain.

**Objectives**

The primary objective of this review is to answer the question: “What key themes emerge from recent literature discussing the potential benefits and limitations of ChatGPT in medical education?” We aim to: (1) Identify the potential applications of ChatGPT in medical education. (2) Assess the challenges and limitations associated with the integration of generative AI. (3) Provide a roadmap for future research on this topic.

**Methods**

This study conducted a scope review, described in accordance with the Preferred Reporting Items for Systematic Reviews and Meta-Analyses Extension for Scoping Reviews (PRISMA-ScR) guidelines [5].

**Eligibility criteria**

We will include articles that: (1) Discuss ChatGPT in the context of medical education. (2) Are written in English. (3) Are published from January 1st, 2022, to November 30th, 2023.

The exclusion criteria included: (1) non-English records; (2) articles that focus solely on non-physician education (such as nursing or dentistry); (3) ChatGPT topics unrelated to medical education; and (4) articles from non-academic sources (e.g., newspapers, internet websites, magazines, etc.).

**Search strategy**

The search strategy will utilize keywords and medical subject headings relevant to chatGPT and medical education. The databases included PubMed/MEDLINE, Embase, and Web of Science.

**Study selection**

The search results from PubMed/MEDLINE, Embase, and Web of Science were imported into EndNote X9 (Clarivate). Article selection was independently conducted by two authors, and discrepancies were resolved through independent review by a third author. A final consensus was reached through author meetings.

**Data extraction**

A specialized search was conducted for each included article, extracting the following information: (1) Article type (preprint, research article, review, commentary, etc.); (2) potential applications and benefits of ChatGPT in medical education; (3) potential risks and limitations of ChatGPT in medical education; and (4) suggestions on the application of ChatGPT in medical education.

**Analysis**

The extracted data underwent thematic analysis. Initially, open coding was performed on the content in the extraction table, followed by the creation of axial codes to categorize existing codes. The data were then recoded into primary and secondary themes decided through discussion. We focused on the potential applications and limitations of ChatGPT in medical education and related suggestions.

**Anticipated outcomes**

We expect to identify the potential applications of ChatGPT in medical education and assess the challenges and limitations associated with the integration of ChatGPT. We aim to provide a foundation for future research and offer feasible insights and evidence for further exploration in this domain.

**Ethics**

This was a literature-based study; therefore, neither approval from the institutional review board nor informed consent was required.

**Conclusion**

This protocol provides a methodology for a scoping review aimed at understanding opportunities, challenges, and future directions of large language models in medical education.

**References**

1. Ghassemi M, Birhane A, Bilal M, Kankaria S, Malone C, Mollick E, Tustumi F. ChatGPT one year on: who is using it, how and why? Nature 2023;624:39-41. <https://doi.org/10.1038/d41586-023-03798-6>

2. Deng J, Lin Y. The benefits and challenges of ChatGPT: an overview. Front Comput Intell Syst 2022;2:81-83. https://doi.org/10.54097/fcis.v2i2.4465

3. Alam F, Lim MA, Zulkipli IN. Integrating AI in medical education: embracing ethical usage and critical understanding. Front Med (Lausanne) 2023;10:1279707. <https://doi.org/10.3389/fmed.2023.1279707>

4. Dave T, Athaluri SA, Singh S. ChatGPT in medicine: an overview of its applications, advantages, limitations, future prospects, and ethical considerations. Front Artif Intell 2023;6:1169595. <https://doi.org/10.3389/frai.2023.1169595>

5. Tricco AC, Lillie E, Zarin W, O’Brien KK, Colquhoun H, Levac D, Moher D, Peters MD, Horsley T, Weeks L, Hempel S, Akl EA, Chang C, McGowan J, Stewart L, Hartling L, Aldcroft A, Wilson MG, Garritty C, Lewin S, Godfrey CM, Macdonald MT, Langlois EV, Soares-Weiser K, Moriarty J, Clifford T, Tuncalp O, Straus SE. PRISMA Extension for Scoping Reviews (PRISMA-ScR): checklist and explanation. Ann Intern Med 2018;169:467-473. <https://doi.org/10.7326/M18-0850>
